# Supplementary figures and images for: New structures of Class II Fructose-1,6-Bisphosphatase from Francisella tularensis provide a framework for a novel catalytic mechanism for the entire class
Source: PLoS One. 2023 Jun 23;18(6):e0274723. doi: 10.1371/journal.pone.0274723 (PMC10289334; doi:10.1371/journal.pone.0274723)

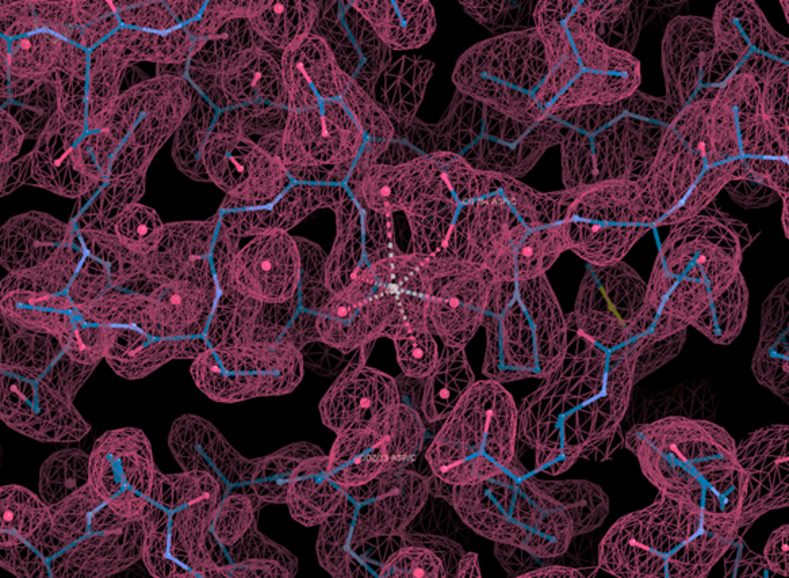

Supplement: S1 Fig — 2Fo-Fc electron density map contoured at 1.5σ. (TIF) [file pone.0274723.s001.tif]

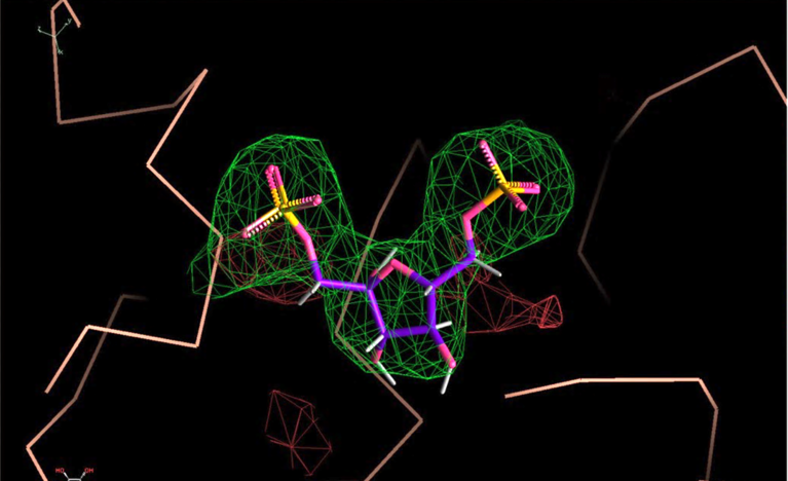

Supplement: S2 Fig — (TIF) [file pone.0274723.s002.tif]

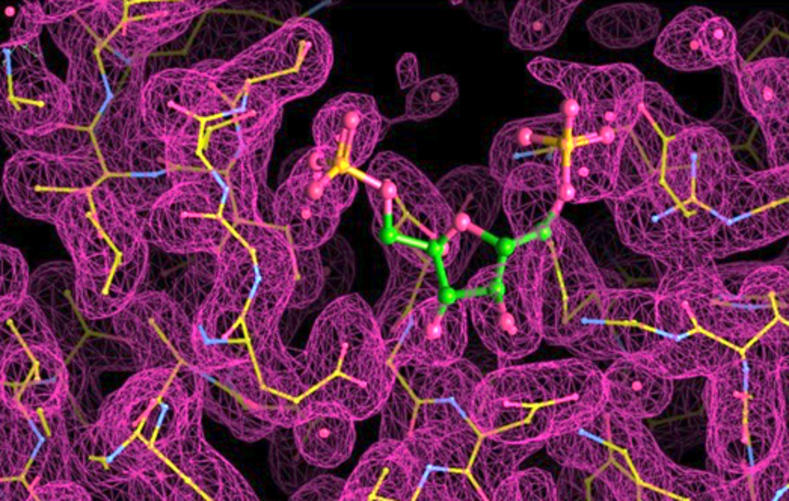

Supplement: S3 Fig — (TIF) [file pone.0274723.s003.tif]
