# Supplementary material for: New structures of Class II Fructose-1,6-Bisphosphatase from Francisella tularensis provide a framework for a novel catalytic mechanism for the entire class
Source: PLoS One. 2023 Jun 23;18(6):e0274723. doi: 10.1371/journal.pone.0274723 (PMC10289334; doi:10.1371/journal.pone.0274723)
Supplement: S1 Table — (DOCX) [file pone.0274723.s004.docx]

| **Type** | **Chain** | **Residue** | **RSCC** | **B-factors (Å^2^)** | **Q<O.9** |
| --- | --- | --- | --- | --- | --- |
| Glycerol | A | 401 | 0.59 | 62,81,83,83 | 0 |
| Glycerol | B | 401 | 0.69 | 53,65,69,76 | 0 |
| Glycerol | B | 403 | 0.77 | 45,62,64,65 | 0 |
| Glycerol | B | 404 | 0.88 | 49,51,57,63 | 0 |
| Glycerol | B | 405 | 0.82 | 48,55,58,60 | 0 |
| Glycerol | C | 601 | 0.78 | 29,45,50,57 | 0 |
| Glycerol | C | 701 | 0.89 | 26, 34,37,39 | 0 |
| Glycerol | D | 601 | 0.78 | 28,43,45,52 | 0 |
| Glycerol | D | 602 | 0.91 | 36,38,42,45 | 0 |
| Glycerol | D | 603 | 0.90 | 35,46,49,51 | 0 |
| Glycerol | D | 604 | 0.85 | 33,45,53,56 | 0 |
| Glycerol | D | 606 | 0.87 | 37,44,48,48 | 0 |
| P04 | B | 402 | 0.96 | 60,62,63,64 | 0 |
| P04 | C | 702 | 0.88 | 33,35,37,39 | 5 |
| P04 | D | 605 | 0.90 | 69,70, 72, 81 | 0 |
| Mn | C | 703 | 0.97 | 52 | 0 |
| Mn | D | 607 | 0.56 | 90 | 0 |
| Mn | A | 402 | 0.85 | 75 | 0 |
| Mn | B | 406 | 0.62 | 78 | 0 |
