# Supplementary material for: New structures of Class II Fructose-1,6-Bisphosphatase from Francisella tularensis provide a framework for a novel catalytic mechanism for the entire class
Source: PLoS One. 2023 Jun 23;18(6):e0274723. doi: 10.1371/journal.pone.0274723 (PMC10289334; doi:10.1371/journal.pone.0274723)
Supplement: Attachment — Submitted filename: Reponse-to-Reviewers.docx [file pone.0274723.s006.docx]

Response to Referees,

My colleagues and I are pleased to submit the final revised version of the manuscript entitled ‘*New structures of Class II Fructose-1,6-Bisphosphatase from Francisella tularensis provide a framework for a novel catalytic mechanism for the entire class’* for your consideration for publication in *PLoS One* as an original research article. Corresponding authors are Farahnaz Movahedzadeh and Celerino Abad-Zapatero.

The revised version addresses the issues raised by one of the referees (#2) as follows. A reference was missing (Nishimasu, 2004-Structure) introducing the characteristics of Class V FBPases. This reference has been added and the corresponding sequence of references revised all through the text and in the final list. The controversial sentence containing ‘Ca-Ca’ distance has been removed. The complete text of this revised version has been carefully reviewed for typos and other minor editorial discrepancies.
